# Selective Isolation of *Bifidobacterium* From Human Faeces Using Pangenomics, Metagenomics, and Enzymology

**DOI:** 10.3389/fmicb.2021.649698

**Published:** 2021-04-21

**Authors:** Shuanghong Yang, Xinqiang Xie, Jun Ma, Xingxiang He, Ying Li, Mingzhu Du, Longyan Li, Lingshuang Yang, Qingping Wu, Wei Chen, Jumei Zhang

**Affiliations:** ^1^School of Food Science and Technology, Jiangnan University, Wuxi, China; ^2^Guangdong Provincial Key Laboratory of Microbial Safety and Health, State Key Laboratory of Applied Microbiology Southern China, Institute of Microbiology, Guangdong Academy of Sciences, Guangzhou, China; ^3^Department of Gastroenterology, First Affiliated Hospital of Guangdong Pharmaceutical University, Guangzhou, China

**Keywords:** *Bifidobacterium*, glycoside hydrolase, metagenomics, pangenomics, enzymology

## Abstract

*Bifidobacterium*, an important genus for human health, is difficult to isolate. We applied metagenomics, pangenomics, and enzymology to determine the dominant glycoside hydrolase (GH) families of Bifidobacterium and designed selective medium for *Bifidobacterium* isolation. Pangenomics results showed that the GH13, GH3, GH42, and GH43 families were highly conserved in *Bifidobacterium*. Metagenomic analysis of GH families in human faecal samples was performed. The results indicated that *Bifidobacterium* contains core GHs for utilizing raffinose, D-trehalose anhydrous, D(+)-cellobiose, melibiose, lactulose, lactose, D(+)-sucrose, resistant starch, pullulan, xylan, and glucan. These carbohydrates as the main carbon sources were applied for selective media, which were more conducive to the growth of bifidobacteria. In the medium with lactose, raffinose and xylan as the main carbon sources, the ratio of cultivable bifidobacteria to cultivable microorganisms were 89.39% ± 2.50%, 71.45% ± 0.99%, and 53.95% ± 1.22%, respectively, whereas the ratio in the ordinary Gifu anaerobic medium was only 17.90% ± 0.58%. Furthermore, the species significantly (*p* < 0.05) varied among samples from different individuals. Results suggested that xylan might be a prebiotic that benefits host health, and it is feasible to screen and isolate bifidobacteria using the oligosaccharides corresponding to the specific GHs of bifidobacteria as the carbon sources of the selective media.

## Introduction

Among the numerous microbial communities that colonise the human body, the intestinal microbiome plays a major role in maintaining host health. The intestinal microbiome of the gastrointestinal ecosystem comprises a collective genome of trillions of microorganisms. Interactions between the host and the intestinal microbiota are complex. Changes in the intestinal microbiota might be critical to prevent or treat various intestinal and non-intestinal diseases (Patel and DuPont, 2015; Ghaisas et al., 2016), such as irritable bowel syndrome (Staudacher et al., 2017), ulcerative colitis (UC), central nervous system diseases, Alzheimer’s disease, and Parkinson’s disease (Sochocka et al., 2019). Colonisation by intestinal microorganisms begins due to differences in microflora between the placenta and amniotic fluid; then, it continues *post-partum via* microorganisms in breast milk (Collado et al., 2016). A key function of the gut microbiota is to facilitate host food digestion, especially that of complex carbohydrates in mammalian diets (Armstrong et al., 2018; Cerqueira et al., 2020). The enzymes involved in polysaccharide decomposition are carbohydrate active enzymes (CAZymes), which consist of carbohydrate-binding modules, carbohydrate esterases, polysaccharide lyases, glycoside transferases, and glycoside hydrolases (GHs), the latter of which are the largest of the CAZymes.

*Bifidobacterium*, a prevalent genus among intestinal microbiota (Mitsuoka, 2014; O’Callaghan and van Sinderen, 2016), is an important symbiotic group, and *Bifidobacterium* genus is one of the first to colonise the human gastrointestinal tract (Turroni et al., 2012; Lewis et al., 2015; O’Callaghan and van Sinderen, 2016). It is believed to exert positive health effects on hosts (Hsieh et al., 2015; Matson et al., 2018). The numbers of bifidobacteria in the total colonic microbiota of normally delivered breast-fed infants decrease from 90% to <5% in adults (Riviere et al., 2016). The abundance of bifidobacteria in the gastrointestinal tract is related to some pathologies, such as intestinal (Grazul et al., 2016; Roškar et al., 2017) and mental (Pinto-Sanchez et al., 2017) diseases. Bifidobacteria can also improve the intestinal barrier function by producing salts of short chain fatty acids such as butyrate (Rios-Covian et al., 2015) and acetate (Hsieh et al., 2015) and reducing serum FITC-dextran levels in a mouse model of colitis (Srutkova et al., 2015). Furthermore, *Bifidobacterium longum* 1714 positively affects cognition in mice (Savignac et al., 2015). In addition to the resistance and regulation of diseases, bifidobacteria can promote nutrient yield to enhance immunity (Kim et al., 2014; Yang et al., 2014; Jaglan et al., 2019).

Tissier isolated the first strain of *Bifidobacterium* from the faeces of exclusively breastfed infants in 1899 (Riviere et al., 2016), and today, the bifidobacteria group includes 85 species^1^. However, the isolation of new *Bifidobacterium* species or their cultivation is difficult because they are strictly anaerobic and easily contaminated by other bacteria. Traditional methods of separation and cultivation no longer meet actual needs, and thus, new technologies are required to explore the taxonomic and functional profiles of microbial DNA extracted from microbial communities, which are constantly increasing (Al-Masaudi et al., 2017). Lugli et al. (2019) proposed that they could mine genetic information of related *Bifidobacterium* strains based on whole metagenome shotgun sequencing to design a reasonable selective medium based on GHs and obtain target strains when screening new *Bifidobacterium* strains. However, they only mined GH based on metagenomic data to design the medium. Monosaccharides are limited in the large intestine, which is inhabited by bifidobacteria. Therefore, bifidobacteria possess various glycosidases and sugar transporters that assimilate indigestible polysaccharides, oligosaccharides, and complex carbohydrates (Garrido et al., 2012). The gene content of the *Bifidobacterium* genome encodes numerous enzymes with predicted roles in carbohydrate modifications (Pokusaeva et al., 2011). To promote the growth of the target genera and inhibit the growth of other microorganisms, selective medium can be designed using omics to identify GHs and their corresponding carbohydrates in samples with unique effects.

We designed 11 selective media based on data derived from pangenomics, metagenomics, and enzymology to promote the proliferation of *Bifidobacterium* while simultaneously reducing the growth of other intestinal bacteria. Stool samples for faecal bacterial transplantation were collected from the First Affiliated Hospital of Guangdong Pharmaceutical University. We also combined the results of a pangenomic analysis of *Bifidobacterium* to suitable GHs to select a suitable carbon source for selectively isolating this genus. The growth of *Bifidobacterium* on different selective media was determined and analysed using 16S amplicon sequencing to determine the optimal carbon source for *Bifidobacterium* isolation.

## Materials and Methods

### Microbial Genome Sequences

We retrieved complete genome sequences for 144 bifidobacteria and 5 other genera (Supplementary Table 2 sheet 6) from the National Center for Biotechnology Information (NCBI) public database. These sequences were used as inputs for the pangenomic analysis to identify specific GHs.

### Collection of Faecal Samples

Stool samples were collected from three healthy donors of faecal microbiota transplantation (FMT) at the First Affiliated Hospital of Guangdong Pharmaceutical University. The study was approved by the ethics committee of the First Affiliated Hospital of Guangdong Pharmaceutical University (reference 2017-98). Healthy donors were recommended to not eat spicy and greasy food at least the day before donating faeces. The donors collected the faeces in a sterile container and provided it to a professional for pre-treatment of the FMT. The pretreatment steps were as follows. Install the faecal collection barrel into the automated faecal bacteria separation system GenFMTer (FMT medical, Nanjing, China), add physiological saline to stir, filter, and purify, which can remove faecal residue and large particles. And then collect the faecal bacteria suspension and repeatedly washed three times. The supernatant was removed, and then resuspended in physiological saline.

S171, S181, and S201 were human stool samples (donor S17, S18, and S20) that had not been processed before FMT; the stool samples S171 and S181 were washed to remove food residue (donor S17 and S18), used for FMT in this hospital, and named as S172 and S182. Approximately 2 g of faeces from the original faeces and 2 g of faeces after pre-treatment were taken.

### DNA Extraction

Total faecal DNA and DNA used for 16S rRNA gene sequencing were extracted using QIAamp PowerFecal Pro FNA Kits (50) (Qiagen GmbH, Hilden, Germany), as per the manufacturer’s instructions. We extracted DNA from *Bifidobacterium* using gram-positive bacterial DNA extraction kits (Magen Biotech, Guangzhou, China). The approximate concentration and purity of all DNA samples were measured using a NanoDrop 2000 spectrophotometer (Thermo Fisher Scientific Inc., Waltham, MA, United States), and concentrations were accurately measured using a Qubit 3.0 fluorometer (Thermo Fisher Scientific Inc.).

### Metagenome Sequencing and Data Analysis

A DNA library was prepared and sequenced using a NovaSeq 6000 system (Illumina, Inc., San Diego, CA, United States) at a commercial laboratory (Novogene Co., Ltd., Beijing, China). Barcodes were removed from the sequencing results using Practical Extraction and Reporting Language (Perl) script, low quality data were removed using Trimmomatic Version 0.39 (Bolger et al., 2014), and host DNA was removed using CLC Genomics Workbench (Qiagen). Thereafter, assembling and announcement were conducted using SPAdes Version 3.12.0 (Nurk et al., 2013) and Prokka (Version 1.13.7) (Seemann, 2014), respectively.

### Pangenomic Analysis

A total of 144 *Bifidobacterium* genome sequences were downloaded from the NCBI database for pan-genome analysis. The sequences were then annotated and compared them with annotation files through a local server. We also annotated the 144 genomes using the dbCAN-seq database. Relative abundance heat maps were prepared using Prism 8.2.1 (GraphPad Software Inc., San Diego, CA, United States), and stack columns of abundance were drawn using OriginPro^®^ 8.5 (OriginLab Corporation, Northampton, MA, United States).

### Designed Selective Medium of *Bifidobacterium*

Traditional GAM (Gifu anaerobic medium) consists of soytone (10 g), proteose peptone (10 g), bovine serum albumin (13.5 g), yeast extract (5 g), beef extract (2.2 g), KH_2_PO_4_ (2.5 g), liver extract (1.2 g), NaCl (3.0 g), L-cysteine (0.3 g), sodium thioglycolate (0.3 g), glucose (3 g), and soluble starch (5 g) in 1 L of water. Moreover, for the solid medium plates, agar was added (15 g/L). The selective medium was designed by eliminating glucose and soluble starch and replacing with raffinose, D-trehalose anhydrous, D(+)-cellobiose, melibiose, lactulose, lactose, D(+)-sucrose, resistant starch, pullulan, xylan, and glucan.

### 16S rRNA Amplicon and Data Analysis

A DNA library was prepared and sequenced using a Nova PE250 instrument (Illumina, Inc., San Diego, CA, United States) at a commercial laboratory (Genewiz, Inc., Suzhou, China). The original binary base-calling data obtained by sequencing were converted into PF (pass filtering) or raw sequence data using Illumina bcl2fastq software. Barcodes were removed from raw data using the Perl script and low-quality data were removed using Trimmomatic version. 0.39 (Bolger et al., 2014). Relative abundance heat maps were prepared using Prism 8.2.1 (GraphPad Software Inc., San Diego, CA, United States) and stack columns of abundance were drawn using OriginPro^®^ 8.5 (OriginLab Corporation, Northampton, MA, United States).

### Isolation and Identification of *Bifidobacterium*

The 11 carbohydrates were selected as the main carbon source, based on all analytical findings (Table 1 and Supplementary Table 1). Fresh faeces samples (0.5 g) were diluted 10^–1^, 10^–2^, 10^–3^, 10^–4^, 10^–5^, and 10^–6^ in physiological saline (0.9%), and then, the 10^–4^, 10^–5^, and 10^–6^ dilutions were coated on the different designed medium plates, with each gradient performed in triplicate for each medium. After incubation at 37°C for 48–72 h in the anaerobic workstation, single colonies were inoculated and streaked on plates and cultured in the anaerobic workstation at 37°C for 48–72 h. This step was repeated. Finally, 16S rRNA gene sequences were amplified from DNA extracts of pure cultures using the primer pair 27F (5′-AGAGTTTGATCCTGGCTCAG-3′) and 1492R (5′-GGTTACCTTGTTACGACTT-3′), which targets the variable region between the 16S rRNA gene sequences. Bifidobacteria were phylogenetically analysed using MEGAX and iTOL^2^.

**TABLE 1:** Structure prediction of selected GHs and corresponding carbohydrates.

GHs		Structure prediction		Carbohydrate
		Structure	Catalytic residues	
Oligo-1,6-glucosidase (EC 3.2.1.10)	GH13	(β/α)_8_ barrel structure, 4 β sheets	Asp212, Pro274, and Ala335	Raffinose, Dextran, Resistant starch
Trehalose-6-phosphate hydrolase (EC 3.2.1.93)	GH13	(β/α)_8_ barrel structure, 6 β sheets	Asp205, Trp267, and Asp329	D-Trehalose anhydrous
Beta-glucosidase (EC 3.2.1.21)	GH3	Consisting of two chains		D(+)-Cellobiose, Melibiose
β-galactosidase (EC 3.2.1.23)	GH42	(β/α)_8_ barrel structure		Lactulose, Lactose
Sucrose phosphorylase (EC 2.4.1.7)	GH13	Consisting of two chains, (β/α)_8_ barrel structure, 4 β sheets		D(+)-Sucrose
Pullulanase (EC 3.2.1.41)	GH13	The N-terminal and C- terminal were composed of 7 β sheets, and a (β/α)_8_ barrel structure in the middle.	Asp343, Glu375, and Asp466	Pullulan
β-xylosidase (EC 3.2.1.37)	GH43	Consisting of two chains; almost all were β sheets		Xylan

## Results

### Pangenomic and Genomic Analyses Identify Specific GHs in *Bifidobacterium*

**FIGURE 1:**
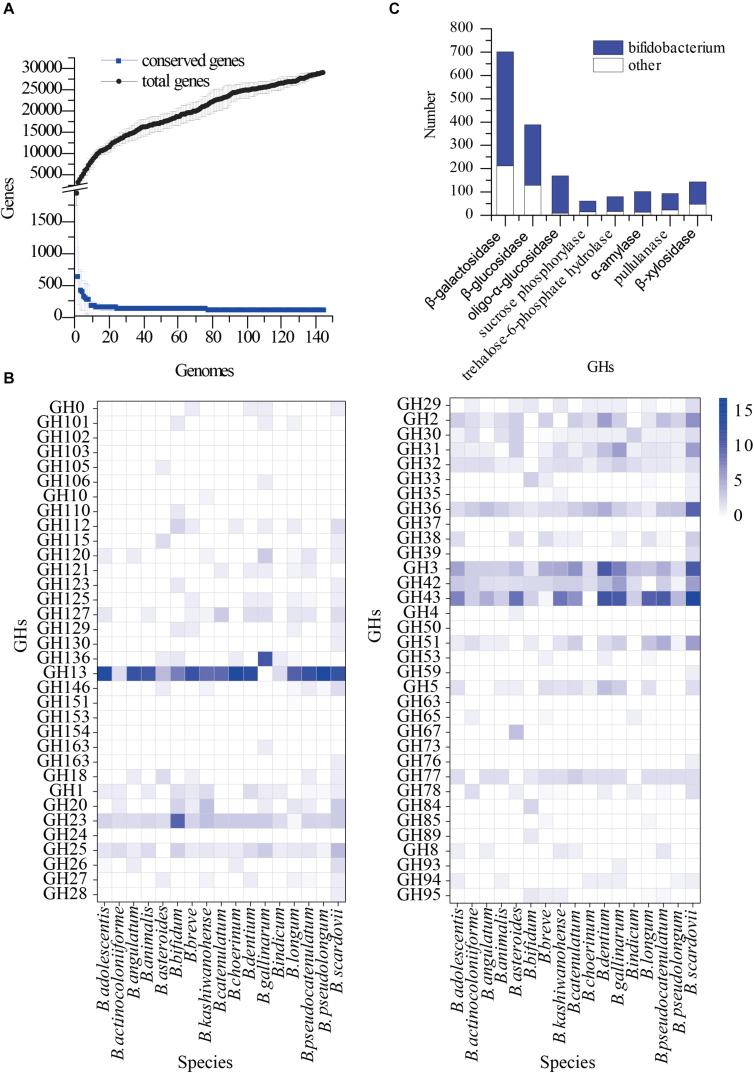
Pangenomics analysis of *Bifidobacterium*. **(A)** The number of conserved genes and total genes as the number of genomes increasing; **(B)** Distribution of glycoside hydrolase families in 144 *Bifidobacterium* genomes, annotated with the dbCAN-seq database; **(C)** Distribution of GH13, GH3, GH42, GH43 in Pangenomics of *Bifidobacterium* and other bacteria.

A total of 144 bifidobacterial genome sequences were downloaded from NCBI for pangenomic analysis (Figure 1A and Supplementary Table 2 sheets 1–3). The total number of genes increased as the genomes increased, whereas conserved genes began to plateau at 20 genomes and remained constant thereafter. We used these whole genomes to find GHs, which are abundant in bifidobacteria species. The glycoside hydrolase annotations of 144 bifidobacteria strains were analysed (Figure 1B and Supplementary Table 2 sheet 5) using the dbCAN-seq database^3^ (Huang et al., 2018). It was found that the GH13, GH3, GH42, and GH43 families were almost all contained and abundant in the *Bifidobacterium* genus. These GH families are involved in the hydrolysis of starch, cellulose, and their related hydrolysed derivatives. Starch, cellulose and hemicellulose are the most common plant polysaccharides and are important sources of energy (Brownlee et al., 2018; Soliman, 2019). They are also an important carbon source for bifidobacteria that inhabit the gastrointestinal tract; therefore, related hydrolytic enzymes may be basically shared by bifidobacteria. They were therefore optimal for finding a suitable carbon source. Among the glycoside hydrolase family, GH43 is the largest branch (Matsuzawa et al., 2015), but these GHs were not the dominant GHs in *Bifidobacterium*, compared with that of other gut microbes. Therefore, we considered the distribution of GHs in other dominant intestinal microorganisms. Based on our previous metagenomic findings of human faeces, we selected 30 complete genomes of five genera, *Alistipes*, *Anaerostipes*, *Barnesiella*, *Blautia*, and *Collinsella* genera, as reference genomes for further pangenomic analysis (Supplementary Table 2 sheet 4) of *Bifidobacterium*. In the previous metagenomic results, the number of this five genera were top few. According to the availability of substrates, economic applicability and the potential as prebiotics, β-galactosidase (EC 3.2.1.23), β-glucosidase (EC 3.2.1.21), oligo-α-glucosidase (EC 3.2.1.10), sucrose phosphorylase (EC 2.4.1.7), trehalose-6-phosphate hydrolase (EC 3.2.1.93), α-amylase (EC 3.2.1.1), pullulanase (EC 3.2.1.41), and β-xylosidase (EC 3.2.1.37) were selected for further analysis. We searched for these enzyme classes in the annotation results in the *Bifidobacterium* pan-genome, and used the results for comparative analysis (Figure 1C). The proportion of these eight enzyme classes in *Bifidobacterium* was significantly higher than other five selected genus. The search results of the carbohydrate active enzyme database and UniProt database were combined to analyse the hydrolysis characteristics of these enzymes (Supplementary Table 1). We further combined the metagenomics results to determine the optimal carbon source for growth.

### Metagenomic Analyses Reveal Specific GHs in Samples

**FIGURE 2:**
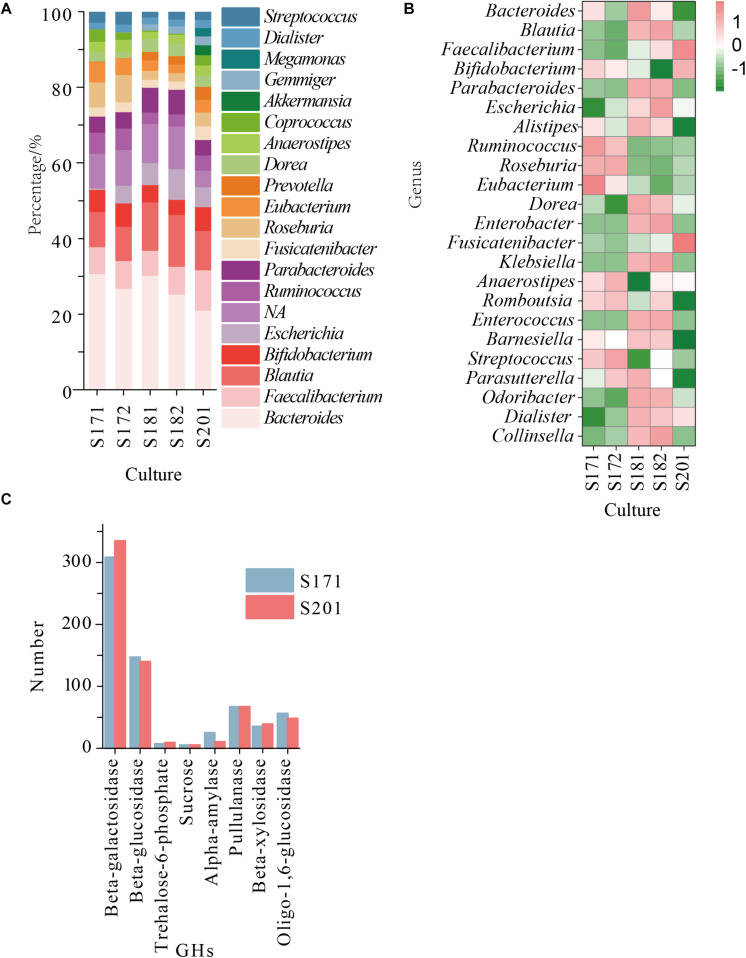
**(A)** The abundance of the reconstructed bacterial genomic material at genus level obtained from sample 17 (S171 and S172), sample 18 (S181 and S182), and sample 20 (S201); **(B)** The relative abundance of the reconstructed bacterial genomic material at genus level obtained from sample 17, sample 18, and sample 20; **(C)** The number of GHs in S171 and S201.

Whole metagenome shotgun sequencing of faecal bacterial samples from transplanted donor stools produced 32–40 million paired reads, with an average approximate length of 150 bp. Species significantly differed among samples from different individuals. The content of *Bifidobacterium* in all samples was in the top five (Figure 2A), and the proportion of *Bifidobacterium* in S201 was the highest, followed by that in S171 and S172, and was lowest in S181 and S182 (Figure 2B). Due to the low abundance of *Bifidobacterium* in S181 and S182, and no significant difference between S171 and S172, in which S172 is the stool after removing food residue from sample S171, only S201 and S171 were used in the next analysis and experiment to screen for bifidobacteria. The pangenomic results of *Bifidobacterium* showed that β-galactosidase, β-glucosidase, trehalose-6-phosphate hydrolase, sucrose phosphorylase, α-amylase, pullulanase, β-xylosidase, and oligo-1,6-glucosidase were abundant in *Bifidobacterium* and the hydrolytic properties of eight specific GHs classes with corresponding organism are shown in Supplementary Table 1. And the amounts of these GHs in the two metagenomic groups were essentially identical (Figure 2C).

Based on the pangenomic and metagenomic analysis, we found several GHs in *Bifidobacterium* that were prominent and important for carbohydrate decomposition (Supplementary Table 1). Therefore, we speculated as to whether oligosaccharides or polysaccharides differ in the selective separation and cultivation of *Bifidobacterium*.

### Selective Medium Designed for *Bifidobacterium* Isolation

**FIGURE 3:**
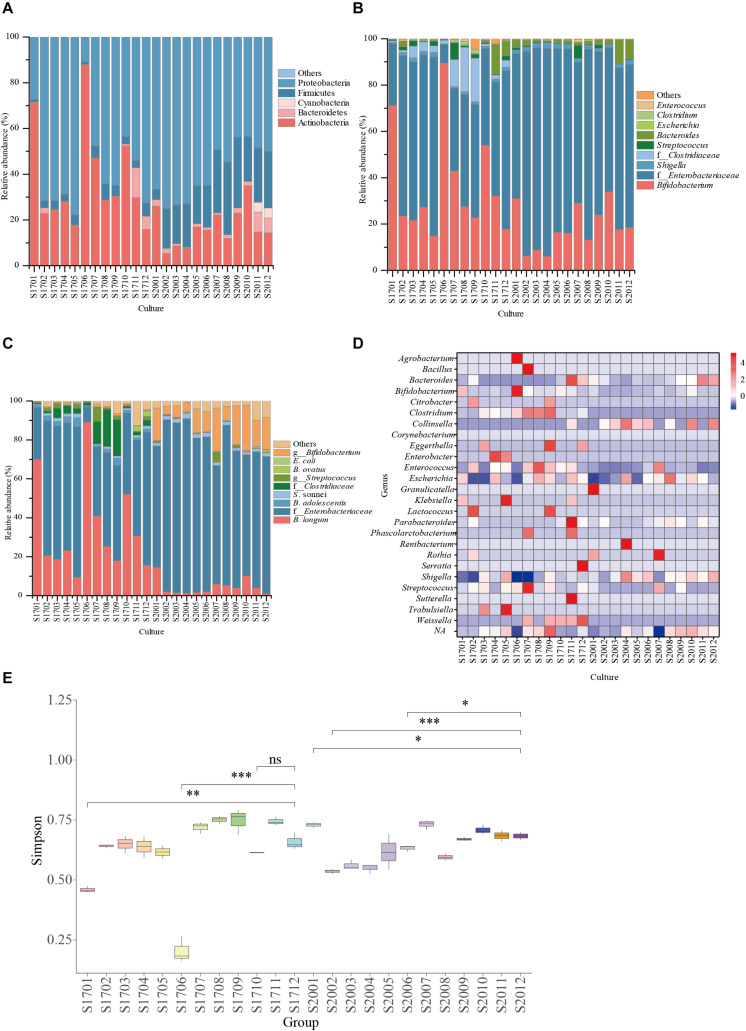
Relative abundance of bacterial community compositions in selective medium plates at family **(A)**, genus **(B)**, or species **(C)** levels; **(D)** The genus-level relative abundance hot map of 16S amplicon sequencing. **(E)** The alpha diversity of 16S amplicon sequencing results. ns, not significant. ^∗^*P* < 0.05, ^∗∗^*P* < 0.01, ^∗∗∗^*P* < 0.001. S1701: S17 represents sample 171 and 01 represents raffinose, 02 represents trehalose, 03 represents cellobiose, 04 represents melibiose, 05 represents lactulose, 06 represents lactose, 07 represents sucrose, 08 represents resistant starch, 09 represents pullulan, 10 represents xylan, 11 represents dextran, and 12 represents the ordinary GAM (glucose and soluble starch). The same is for S2001–S2012, only S20 represents sample 201.

As shown in Table 1, the raffinose, D-trehalose anhydrous, D(+)-cellobiose, melibiose, lactulose, lactose, D(+)-sucrose, resistant starch, pullulan, xylan, and glucan were selected according to the hydrolysis characteristics of the selected GHs. Based on the results of 16S amplicon sequencing of clones collected from sample S171 that were cultured on selective media, in the medium with lactose, raffinose, and xylan as the main carbon sources, the ratio of cultivable bifidobacteria to cultivable microorganisms were 89.39 ± 2.50%, 71.45 ± 0.99%, and 53.95 ± 1.22%, respectively, whereas for the ordinary GAM, it was only 17.90 ± 0.58% (Figure 3, Table 2, and Supplementary Table 2 sheet 7). Except for that in the medium with lactulose as the carbon source, the relative abundance of *Bifidobacterium* in the remaining 10 media was higher than that in ordinary GAM (Figure 3B). However, only half of S201 achieved this result, and the metagenomic results revealed more *Bifidobacteria* in S201 than in S171 (Figure 2B), and the abundance of enzymes in GH classes was basically the same (Figure 2C). The heat map (Figure 3D) also showed significantly higher abundances of *Bifidobacterium* in S171 than in S201, and lower abundances of *Shigella* in S171. Lactose had the best selectivity for *Bifidobacterium*, followed by raffinose in S171, and the selectivity of lactulose was the worst. Xylan was more effective than glucan. Raffinose and xylan exhibited the best selectivity for S201.

**TABLE 2:** Cultivable microorganisms and *Bifidobacterium* on selective medium.

Selective medium	Cultivable microorganisms	Cultivable *Bifidobacterium*	Proportion of *Bifidobacterium* (%)
S1701	21008 ± 1257	4992 ± 824	71.45 ± 0.99
S1702	8124 ± 111	635 ± 69	23.39 ± 1.62
S1703	8395 ± 202	606 ± 62	21.69 ± 1.70
S1704	8604 ± 570	786 ± 486	25.39 ± 11.20
S1705	8596 ± 344	427 ± 109	14.72 ± 2.37
S1706	51616 ± 1367	15404 ± 1569	89.39 ± 2.50
S1707	13639 ± 579	1954 ± 316	42.79 ± 1.60
S1708	10040 ± 257	924 ± 120	27.58 ± 2.81
S1709	12317 ± 713	935 ± 96	23.30 ± 3.80
S1710	12932 ± 343	2329 ± 230	53.95 ± 1.22
S1711	10758 ± 264	1151 ± 233	31.79 ± 4.50
S1712	8298 ± 165	496 ± 45	17.90 ± 0.58
S2001	8618 ± 442	894 ± 235	30.65 ± 3.45
S2002	6396141	134 ± 71	6.08 ± 3.08
S2003	6352 ± 276	186 ± 47	8.67 ± 1.21
S2004	7148 ± 145	145 ± 53	6.22 ± 2.52
S2005	7309 ± 140	400 ± 211	16 ± 8.29
S2006	7511 ± 223	403 ± 71	16.04 ± 2.16
S2007	7665 ± 119	742 ± 146	28.91 ± 4.68
S2008	8109 ± 13	356 ± 27	13.17 ± 1.08
S2009	10149 ± 220	811 ± 94	23.90 ± 1.29
S2010	11817 ± 223	1338 ± 354	33.57 ± 7.14
S2011	9481 ± 150	561 ± 39	17.75 ± 0.52
S2012	8897 ± 248	546 ± 129	18.21 ± 2.04

*S1701: S17 represents sample 171 and 01 represents raffinose, 02 represents trehalose, 03 represents cellobiose, 04 represents melibiose, 05 represents lactulose, 06 represents lactose, 07 represents Sucrose, 08 represents resistant starch, 09 represents Pullulan, 10 represents xylan, 11 represents dextran, and 12 represents the ordinary GAM (glucose and soluble starch). The same is for S2001–S2012, only S20 represents sample 201.*

### Pure Culture Identification

**FIGURE 4:**
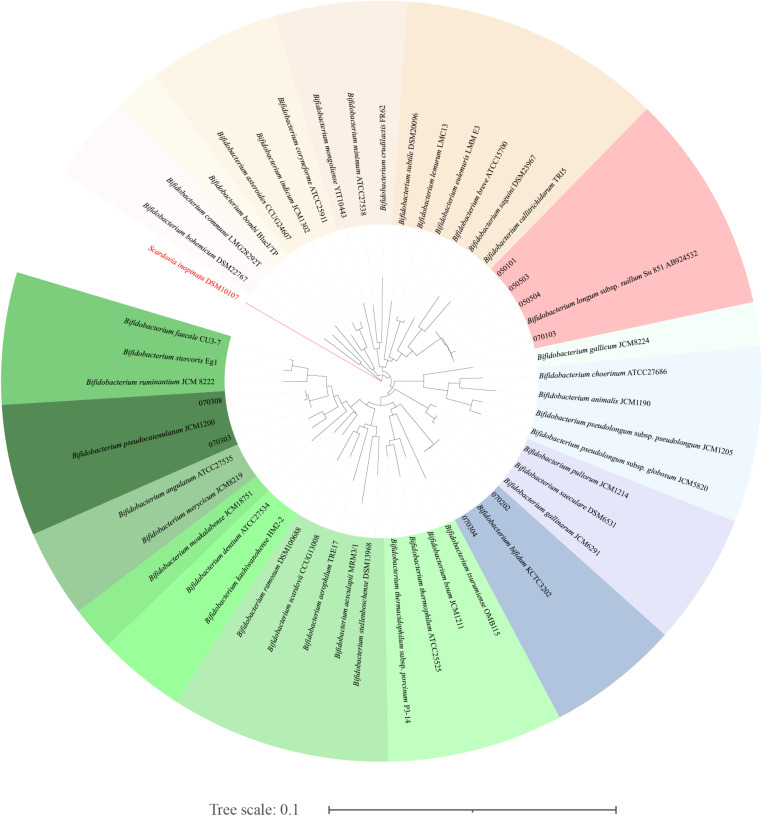
The phylogenetic analysis of the obtained *Bifidobacterium.* The phylogenetic tree was constructed by the neighbour-joining method, with the genome sequence of *Scardovia inopinata* JCM 12537 as outgroup, based on 1000 replicates of the phylogenetic tree.

The results of 16S amplicon sequencing showed that the bacterial composition significantly differed between the S171 and S201 faecal samples. We investigated the details of *Bifidobacterium* from different sources based on 76 colonies. We then purified these colonies and assessed 16S rDNA. We identified 28 (37%) *Bifidobacterium* strains (Table 3), and Figure 4 shows the results of the phylogenetic analysis. These strains belong to the three branches of *B. longum*, *Bifidobacterium pseudocatenulatum*, and *Bifidobacterium bifidum*.

**TABLE 3:** The 16S rDNA identification of selected and predicated colonies.

Genus	Species	Sample 17	Sample 20
*Bacillus*	*paramycoides*	1	0
*Bifidobacterium*	*bifidum*	0	13
	*longum*	8	3
	*pseudocatenulatum*	0	4
*Enterococcus*	*faecalis*	2	0
	*faecium*	0	13
*Escherichia*	*coli*	1	4
	*fergusonii*	10	4
	sp.	0	1
*Glutamicibacter*	sp.	0	1
*Proteus*	*mirabilis*	2	0
*Shigella*	*flexneri*	1	0
*Streptococcus*	*intermedius*	1	0
	*salivarius*	4	2
	sp.	1	0

## Discussion

Many studies have associated gut microbes with human health. Bifidobacteria among intestinal microbes are beneficial microorganisms (Jaglan et al., 2019) and have attracted the most attention. However, strict requirements for growth have caused difficulties with their isolation and purification. The carbon source is an indispensable factor for the growth of microorganisms, and it can be used to screen these bacteria. The distribution of glycoside hydrolases in *Bifidobacterium* as the key enzymes for carbohydrate hydrolysis is important for bacterial culture and functional analysis. Therefore, pangenomic analysis of the whole genome sequence of 144 *Bifidobacterium* species downloaded from NCBI and the dbCAN-seq database revealed the common GH families, GH13, GH3, GH42, and GH43, in *Bifidobacterium*.

The abundance of *Bifidobacterium* obviously differed among samples, and it was the most abundant in S201. The pangenomic findings of *Bifidobacterium* showed that GH13, GH3, GH42, and GH43 were abundant, and the abundance of enzymes in GH classes was basically the same in the metagenomic results of S171 and S201. Based on the hydrolysis characteristics of these enzymes, the corresponding carbohydrates (Table 1) were selected as main carbon source of the selective medium. The abundance of bifidobacteria in sample S201 is higher, so the abundance of bifidobacteria on the solid medium of S201 should be higher, but in fact sample S171 has a higher cultivable *Bifidobacterium* abundance on almost selective plates.

The selectivity of raffinose and xylan for bifidobacteria in both samples was effective based on the 16S amplicon sequencing analysis. Lactose was significantly selective for S171. Not only did the number of bifidobacteria surpass that of the control group (the ordinary GAM), but few other bacterial strains proliferated. Raffinose is an established prebiotic (Ose et al., 2018). Adding raffinose to the medium increases the production of short-chain fatty acids and carbon dioxide, and reduces the final pH and ammonia concentration in the medium (Amorim et al., 2020). In addition, raffinose can increased the relative abundance of probiotics (*p* < 0.05), and decreased that of pathogenic bacteria (Pacifici et al., 2017). Therefore, xylan might also have similar or even more powerful prebiotic effects, but this needs to be further verified by animal experiments and high-performance liquid chromatography (HPLC). Animal experiments were done to verify whether xylan can regulate the intestinal microbiota of mice, i.e., whether there is a difference between the intestinal flora of mice on a diet containing xylan and a diet without xylan and whether mice fed on a xylan-containing diet have more probiotics in their gut microbiota than mice on a xylan-free diet (Pacifici et al., 2017). HPLC was used to determine short-chain fatty acids in the mouse faeces (Li et al., 2020). GH3 and GH43 families containing xylanases and xylan was easily used, thus GH43 or GH3 glycoside hydrolases might be functioning as xylanases in *Bifidobacterium*. The results from the present study were obtained using a limited number of human faecal samples. This work on selective media could benefit further from testing on a larger number of human faecal samples.

## Conclusion

Glycoside hydrolase 13, GH3, GH42, and GH43 are prevalent in *Bifidobacterium*, and the corresponding carbohydrates substrate can serve as the main carbon source in medium to selectively isolate and cultivate *Bifidobacterium*. Xylan might be a prebiotic that benefits host health, but its effects might differ among individuals. Metagenomics and pangenomics allow the accumulation of more information that can facilitate the isolation and cultivation of *Bifidobacterium*. The abundance *of Bifidobacterium* in samples might not mean that more bifidobacteria can be isolated, but it also depends on the characteristics of the *Bifidobacterium* species in samples. In the future, we will further verify the properties of specific GHs and the prebiotic properties of related oligosaccharides.

## Data Availability Statement

The datasets presented in this study can be found in online repositories. The names of the repository/repositories and accession number(s) can be found below: NCBI: BioProject ID PRJNA695860 for 16S rDNA amplicon sequencing and PRJNA695407 for Metagenome.

## Ethics Statement

The studies involving human participants were reviewed and approved by the Ethics Committee of The First Affiliated Hospital of Guangdong Pharmaceutical University (reference 2017-98). The patients/participants provided their written informed consent to participate in this study.

## Author Contributions

JZ, WC, XX, and QW conceived and designed the experiments. XX, SY, MD, LL, and LY conducted the experiments. SY, JM, XH, and YL participated in data collection and processing. SY and XX drafted the manuscript. JZ and XX reviewed the manuscript. All authors read and approved submission of the final version of the manuscript for publication.

## Conflict of Interest

The authors declare that the research was conducted in the absence of any commercial or financial relationships that could be construed as a potential conflict of interest.

## Footnotes

1 https://lpsn.dsmz.de/genus/bifidobacterium

2 iTOL: Interactive Tree Of Life (embl.de)

3 http://www.cazy.org
