# Supplementary material for: Selective Isolation of Bifidobacterium From Human Faeces Using Pangenomics, Metagenomics, and Enzymology
Source: Front Microbiol. 2021 Apr 21;12:649698. doi: 10.3389/fmicb.2021.649698 (PMC8096985; doi:10.3389/fmicb.2021.649698)
Supplement: Supplementary file 1 [file Table_1.DOCX]

Table 1 The hydrolytic properties of 18 specific GHs and their corresponding organism

| GHs | | Function | Organism |
| --- | --- | --- | --- |
| Beta-galactosidase | GH42 | Hydrolysis of terminal non-reducing beta-D-galactose residues in beta-D-galactosides. | *Bifidobacterium adolescentis* |
| Beta-glucosidase | GH3 | Hydrolysis of terminal, non-reducing beta-D-glucosyl residues with release of beta-D-glucose | *Bifidobacterium asteroides* |
| Oligo-1,6-glucosidase | GH13 | Catalysis of the hydrolysis of (1->6)-alpha-D-glucosidic linkages in some oligosaccharides produced from starch and glycogen by alpha-amylase, and in isomaltose. Releases a free alpha-D-glucose (GO - Molecular function) | *Bifidobacterium pseudocatenulatum* |
| Sucrose phosphorylase |  | Catalyzes the reversible phosphorolysis of sucrose into alpha-D-glucose 1-phosphate (Glc1P) and D-fructose | *Bifidobacterium adolescentis* |
| Trehalose-6-phosphate hydrolase |  | Catalysis of the reaction: alpha,alpha-trehalose 6-phosphate + H_2_O = D-glucose + D-glucose 6-phosphate. | *Bifidobacterium animalis* subsp. *lactis* |
| Alpha-amylase |  | Endohydrolysis of (1->4)-alpha-D-glucosidic linkages in polysaccharides containing three or more (1->4)-alpha-linked D-glucose units. | *Bifidobacterium pseudocatenulatum* |
| Pullulanase |  | Endohydrolysis of (1->4)-alpha-D-glucosidic linkages in polysaccharides containing three or more (1->4)-alpha-linked D-glucose units (1,4-alpha-D-glucan hydrolase) | *Bifidobacterium breve* |
| Beta-xylosidase | GH3, 43 | Catalysis of the hydrolysis of (1->4)-beta-D-xylans so as to remove successive D-xylose residues from the non-reducing termini (GO - Molecular function) | *Bifidobacterium scardovii* |
